# Magnetic Nanoemulsions for the Intra-Articular Delivery of Ascorbic Acid and Dexamethasone

**DOI:** 10.3390/ijms241511916

**Published:** 2023-07-25

**Authors:** Camelia Mihaela Zară-Dănceanu, Cristina Stavilă, Anca Emanuela Minuti, Luminiţa Lăbușcă, Valentin Nastasa, Dumitru-Daniel Herea, Răzvan-Nicolae Malancus, Daniel Ghercă, Sorin-Aurelian Pasca, Horia Chiriac, Mihai Mares, Nicoleta Lupu

**Affiliations:** 1 Department of Magnetic Materials and Devices, National Institute of Research and Development for Technical Physics, 700050 Iaşi, Romania; cdanceanu@phys-iasi.ro (C.M.Z.-D.); cstavila@phys-iasi.ro (C.S.); hchiriac@phys-iasi.ro (H.C.);; 2 Faculty of Physics, Alexandru Ioan Cuza University, 700506 Iaşi, Romania; 3 County Emergency Hospital Saint Spiridon, Orthopedics and Traumatology Clinic, 700111 Iaşi, Romania; 4 Faculty of Veterinary Medicine, “Ion Ionescu de la Brad” University of Life Sciences (IULS), 8 Mihail Sadoveanu Alley, 700489 Iaşi, Romaniaspasca@uaiasi.ro (S.-A.P.); mmares@uaiasi.ro (M.M.)

**Keywords:** nanoemulsion, magnetic nanoparticles, magnetic nanoemulsion, adipose derived mesenchymal cells, chondrogenesis, biocompatibility, wound healing

## Abstract

(1) Osteoarthritis (OA) is a progressive joint degenerative disease that currently has no cure. Limitations in the development of innovative disease modifying therapies are related to the complexity of the underlying pathogenic mechanisms. In addition, there is the unmet need for efficient drug delivery methods. Magnetic nanoparticles (MNPs) have been proposed as an efficient modality for the delivery of bioactive molecules within OA joints, limiting the side effects associated with systemic delivery. We previously demonstrated MNP’s role in increasing cell proliferation and chondrogenesis. In the design of intra-articular therapies for OA, the combined NE-MNP delivery system could provide increased stability and biological effect. (2) Proprietary Fe_3_O_4_ MNPs formulated as oil-in-water (O/W) magneto nanoemulsions (MNEs) containing ascorbic acid and dexamethasone were tested for size, stability, magnetic properties, and in vitro biocompatibility with human primary adipose mesenchymal cells (ADSC), cell mobility, and chondrogenesis. In vivo biocompatibility was tested after systemic administration in mice. (3) We report high MNE colloidal stability, magnetic properties, and excellent in vitro and in vivo biocompatibility. By increasing ADSC migration potential and chondrogenesis, MNE carrying dexamethasone and ascorbic acid could reduce OA symptoms while protecting the cartilage layer.

## 1. Introduction

Osteoarthritis (OA) is a multifactorial joint degenerative disease that aggravates in time. The natural course of OA is commonly associated with progressive pain and stiffness, representing a worldwide leading cause of musculoskeletal disability [1]. OA incidence and prevalence is rapidly increasing at a global level due to increasing populational ageing and widespread risk factors such as metabolic disease, obesity, sedentarism, and traffic and war injuries that expose post-traumatic OA [2]. Increasing pain that eludes current available therapies is one of the main factors that progressively deteriorate the quality of life of OA patients and their caregivers, and burdens health care systems [3]. Available systemic non-steroidal drugs are known to elicit important side effect when used as long-term therapies, being very often contraindicated, especially in elderly patients with multiple comorbidities such as renal or cardiac failure [4]. Opioid use has important side effects, does not consistently ameliorate the symptoms, tolerability is low, and efficacy in terms of reducing disability is not clinically relevant in controlled studies [5]. Existent clinical guidelines recommend intra-articular delivery of steroids as a modality for reducing pain and stiffness in moderate to advanced OA [6]. Concerns exist, however, regarding the chondrotoxicity of locally derived corticosteroids [7], especially when associated with local anesthetics [8]. Improved methods of local delivery are expected to consistently reduce toxicity and increase the bio-availability of locally delivered steroids while ideally protecting or at least not contributing to further cartilage damage [9,10].

Magnetic nanoparticles (MNPs) have emerged as a versatile tool for multiple applications in biology and medicine. MNPs can be formulated in a large variety of shapes and coatings and can be functionalized for the delivery of a large panel of drugs or bioactive molecules [11]. Excellent biocompatibility in vitro and in vivo as well as the responsiveness to applied magnetic fields recommend MNPs as promising tools for guided and targeted delivery [12,13]. The excellent stability, long circulation time, and very limited leakage of encapsulated molecules, combined with excellent bioavailability, recommends their extended clinical use [14]. Nanoemulsions consisting of nanosized lipid droplets (NEs) are mainly composed of a dispersed or hydrophobic core, a continuous phase (hydrophilic), and surfactants or stabilizers. Their core can serve as a deposit for hydrophobic materials, while salts or solvents are contained within the hydrophilic phase [15]. Such formulations can be tailored to display on the surface, with targeting or immune evading compounds being recently used for developing groundbreaking mRNA vaccines [16]. NEs with long-term stability improve the bioavailability of lipophilic [17] or hydrophilic drugs [18]. NEs penetrate tissues and are up-taken by mammalian cell membranes, making them suitable for targeted delivery in the case of difficult to reach tissues or cell organelles, trans passing blood–brain barrier [19], or targeting mitochondria [20].

The combination of MNPs and NEs has been proved to retain the advantages of both delivery systems. MNPs’ colloidal stability as well as carrier proprieties can be consistently increased by the addition of lipid formulations. The presence of an iron core can improve targeting, tracking, and on demand delivery features to increase the performance as well as the therapeutic potential of nano lipid formulation [21].

We have previously demonstrated a consistent increase in cell viability as well as in stem cell chondrogenesis in the presence of as-prepared proprietary MNPs [22]. Here we are reporting a magnetic nanoemulsion-based (MNE) drug delivery system based on a MNP core and nano lipid formulation. MNEs carrying clinically approved, commonly used drugs: anti-inflammatory corticosteroid—dexamethasone—and ascorbic acid were tested for in vitro and in vivo biocompatibility after intraperitoneal injection in adult BALB C mice. Their efficiency in supporting adipose derived mesenchymal cells (ADSC) chondrogenesis as well as in promoting cellular motility crucial for regenerative processes was further investigated in vitro.

## 2. Results

### 2.1. Physical Characterization of Magnetoemulsions

#### 2.1.1. Stability, DLS, ζ-Potential Measurements, and Magnetization

The physical stability of MNEs and OA-MNPs was assessed visually immediately after synthesis and periodically, up to five months. Samples were kept at room temperature (20–25 °C). No signs of aggregation, flocculation, or creaming were observed during follow up. In terms of size, the mean diameter of the samples was below 200 nm, namely: 116.63 nm for OA-MNP, 186.25 nm for MNEs-Dex, and 200 nm for MNEs-As.Ac.

Zeta potential was performed in order to evaluate suspension stability via surface charge. Zeta potential recordings displayed values of −148.7 mV for OA-MNP, 20.69 mV for MNEs-Dex, and 20.17 mV for MNEs-As.Ac., proving excellent stability for OA-MNP emulsion and an incipient instability for MNEs (Table 1). A zeta potential higher than 60 mV is a marker of excellent physical stability, indicating that the risk for particle aggregation is extremely low. Negative values for the OA-MNP emulsion were expected due to their oleic acid coating. The high zeta potential value obtained for the OA-MNP emulsion is an indicator of the contribution of the ionized interfacial oleic acid layer to the electrostatic surface charge [23].

#### 2.1.2. MNE Magnetization

**Figure 1:**
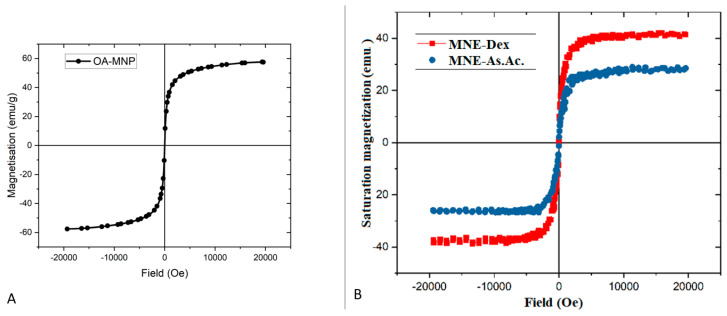
Hysteresis curve obtained at room temperature of OA−MNP (**A**), MNE−Dex (red line), and (**B**) MNE−As.Ac. (blue line).

The OA-MNPs used in the synthesis of magnetic nanoemulsions showed superparamagnetic behavior with a coercivity field of 5.6427 Oe and a saturation magnetization of 57.626 emu/g. The MNEs maintained the magnetic properties with a saturation magnetization of 468 × 10^−6^ emu, and coercive field is 9.0641 Oe for MNEs-Dex; in the case of magnetic MNEs–As.Ac, the saturation magnetization was approximately 290 × 10^−6^ emu, and the coercive field was 8.8285 Oe. The aspect of the hysteresis curve indicates that both MNE samples have a superparamagnetic behavior that falls within the range of detectability of clinical available imagistic methods (e.g., magnetic resonance imaging—MRI) [18] (Figure 1).

#### 2.1.3. MNE Stability

**Figure 2:**
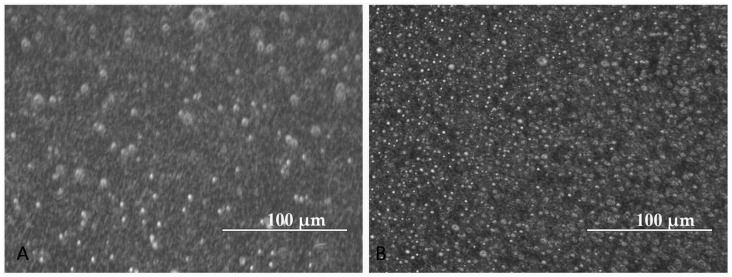
MNE morphological aspects (Inverted microscope, EVOS FL Life Technologies) of (**A**) MNE-Dex and (**B**) MNE-As.Ac. demonstrate nanoemulsion stability after 14 days.

**Figure 3:**
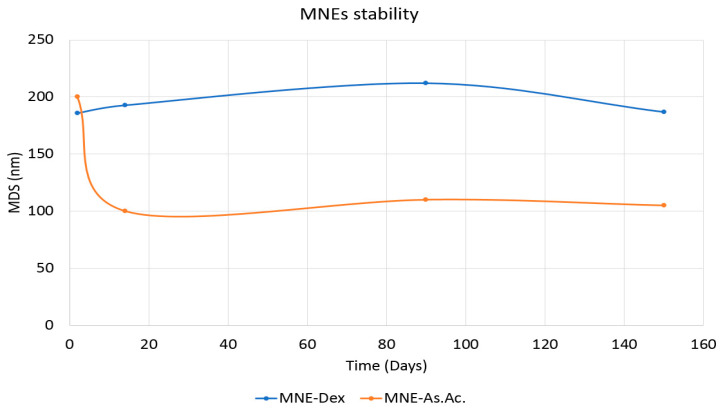
MNE mean size diameter measured at 2, 14, 90, and 150 days for MNE-Dex (Blue) and MNE-As.Ac. (Orange) using dynamic light scattering (DLS).

The morphological aspects at 14 days after preparation of MNEs-Dex and MNEs-As.Ac. are presented in Figure 2, demonstrating uniform distribution without clogging and agglomerates. It is important to note that MNEs-Dex and MNEs-As.Ac. remained stable for at least 5 months, as shown by the graph of mean size diameter up to 150 days (Figure 3).

#### 2.1.4. MNE In Vitro Drug Release

**Figure 4:**
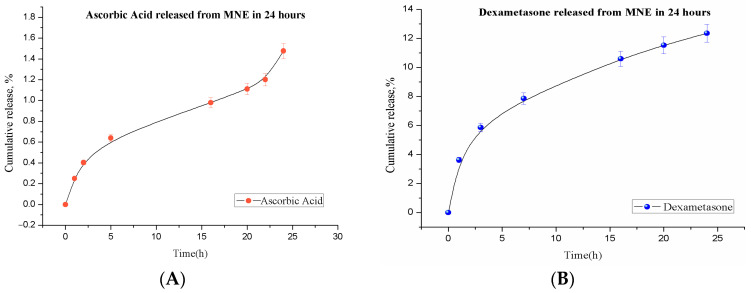
Kinetics of in vitro release from MNE over 24 h: (**A**) ascorbic acid release; (**B**) dexamethasone release obtained by dialysis membrane method over 24 h.

MNEs were tested as potential drug delivery systems for the gradual release of dexamethasone and ascorbic acid. In vitro release performed on dialysis membranes over 24 h revealed slow and constant release for both loaded drugs; 12.2% of dexamethasone total payload was released from MNEs-Dex, while only 1.5% of total As.Ac was delivered after 24 h in this in vitro system (Figure 4A,B).

### 2.2. Magnetoemulsion Interaction with Adipose Derived Mesenchymal Cells—ADSC

#### 2.2.1. Morphological aspects of MNEs upload in ADSC

**Figure 5:**
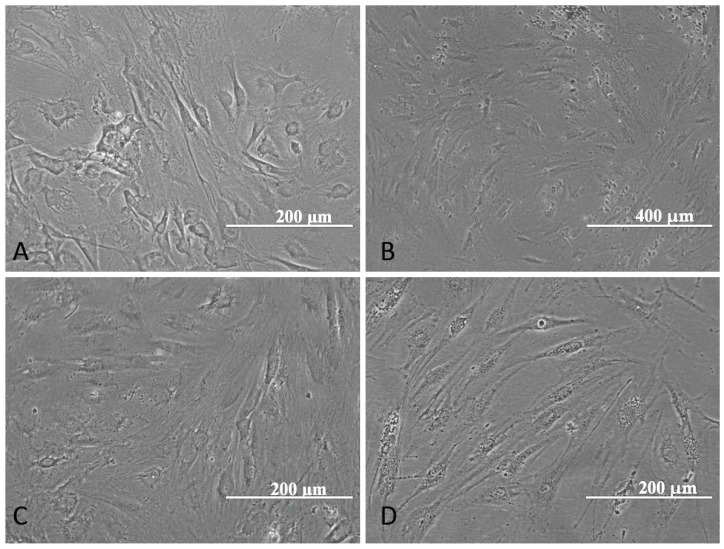
ADSC co-incubated with MNEs: morphological aspect of ADSC without MNEs (**A**), ADSC loaded with OA-MNP (**B**), ADSC with MNEs-Dex (**C**), and ADSC with MNEs-As.Ac. (**D**).

ADSCs incubated for 48 h with 10% MNEs added to CM were shown to retain the normal structure of cellular bodies, without apparent cellular membrane interruption (Figure 5C,D). MNEs were observed within the cytoplasm and around cell membranes, even after 24 h of incubation. A difference in particle distribution can be observed between AO-MNPs (Figure 5B) and as-prepared MNEs (Figure 5C,D); MNEs appear to be more evenly distributed inside the cell cytoplasm and protruding dendrites in comparison to AO-MNPs.

#### 2.2.2. Iron Cellular Uptake

**Figure 6:**
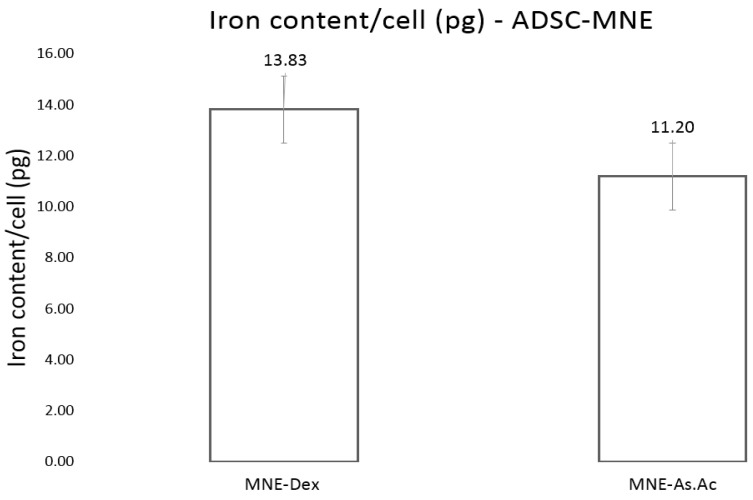
Iron content per cellular element in adipose-derived mesenchymal cells detected using ferrozine assay at 24 h for MNE-Dex and MNE-As.Ac. Results expressed after subtracting cellular iron amount of non-loaded ADSC controls.

Addition of MNEs to ADSC culture by simple dispersion in culture media resulted in in iron uptake of average 13.8 and 11.2 pg/cell for MNEs-Dex and MNEs-As.Ac, respectively, after 48 h cell-MNE interaction. The amount of iron upload recorded here corresponds to the lower range of iron content that allows traceability using MRI and to good traceability using magnetic particle imaging (MPI) [19] (Figure 6).

### 2.3. Magnetoemulsion Biocompatibility

#### 2.3.1. In Vitro Cytotoxicity on ADSC

**Figure 7:**
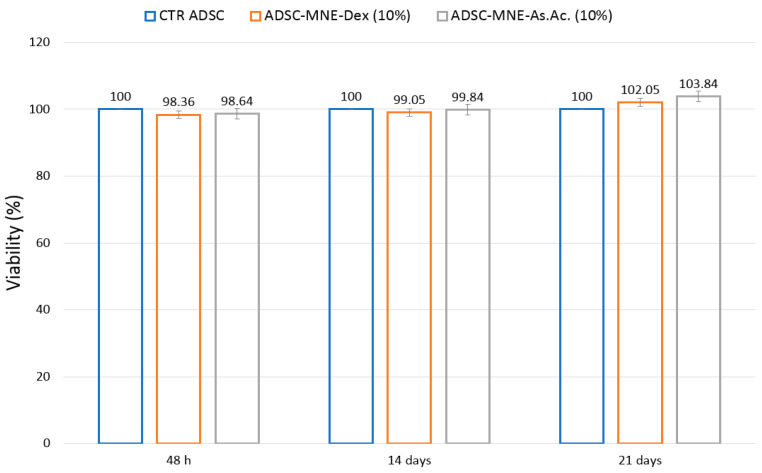
Cell viability (MTT assay) at 48 h and 14 and 21 days: MNE-Dex = magneto-nanoemulsions with dexamethasone; MNE-As.Ac. = magneto-nanoemulsions with ascorbic acid; ADSC = adipose-derived mesenchymal cells; percentual cell viability calculated in reference to non-treated controls for each cell type.

In vitro MNE cytotoxicity was assessed using a tetrazolium salt assay (MTT test). The assay is an indicator of mitochondrial activity in tested cells and revealed that at both short time (48 h) as well as medium and long time (7 and 21 days, respectively) cell viability is not affected by MNE presence (Figure 7).

**Figure 8:**
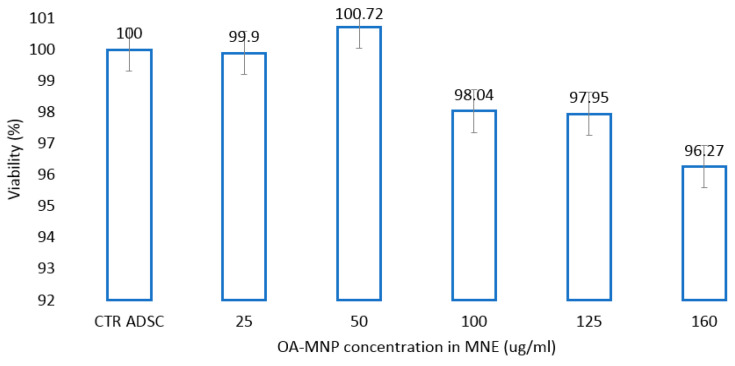
Cell viability (MTT assay) at different concentration (25, 50, 75, 100, 125 (µg/mL)) of magnetic nanoparticles contained by MNE; ADSC = adipose-derived mesenchymal cells; MNE-Dex = magneto-nanoemulsions with dexamethasone; MNE-As.Ac. = magneto-nanoemulsions with ascorbic acid.

A slight, non-significant increase in cell viability witnessing cell proliferation was detected in the presence of MNEs-As.Ac. ADSCs exposed to an increasing concentration of MNEs as calculated by the amount of AO-MNPs per MNEs suspension do not significantly decrease viability compared to controls at loading concentrations up to 160 µg/mL (Figure 8).

#### 2.3.2. In Vivo MNE Biocompatibility

**Figure 9:**
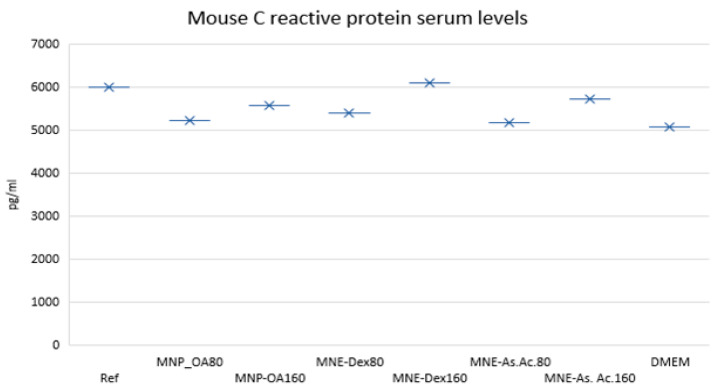
Mouse C reactive protein serum levels at two concentrations (80 and 160 µg/mL) of MN contained by MNE for MNP-OA, MNE-Dex, and MNE-As.Ac.

After intraperitoneal injection of two doses of MNE suspension (300 μL of 80 and 160 µg/mL suspension, respectively) mice were observed daily. Seven days after injection, there was no change in red blood (Table 2) cell count (RBC), white blood cell count (WBC), hemoglobin, hematocrit, or platelets compared to controls. There was no significant alteration in the WBC distribution profile compared to controls (*p* > 0.05), with parameter variations within the physiological range of BALB C mice (Supplementary Material, Table S1). The ELISA test for detection of circulating CRP from serum samples revealed no significant variability compared to control mice injected with similar amount of saline and relative to laboratory reference values (Figure 9).

**Figure 10:**
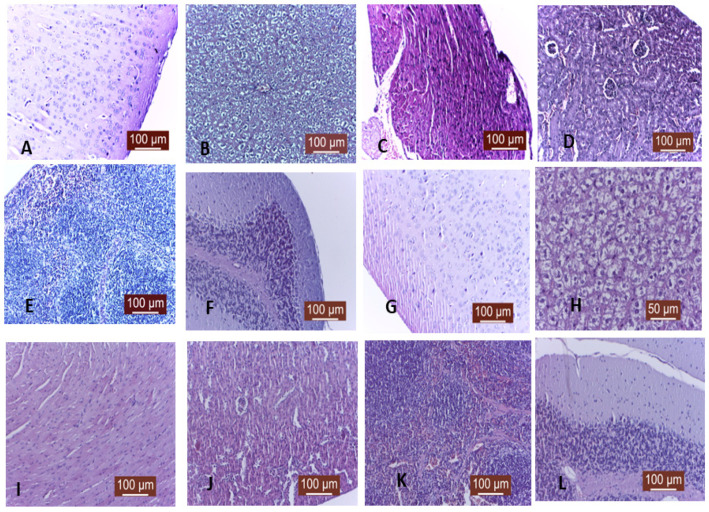
Morphological aspect of several target organs in mice after intraperitoneal injection of a large dose (300 microliter) of MNE-Dex (**A**–**F**) and MNE-As.Ac. (**G**–**L**), 160 µg/mL, using a Masson trichrome staining Inverted microscope: (**A**) brain tissue; (**B**) liver tissue; (**C**) myocardium; (**D**) kidney; (**E**) spleen. (**F**) cerebellum; (**G**) brain; (**H**) liver tissue; (**I**) myocardium; (**J**) kidney; (**K**) spleen; (**L**) cerebellum. There were no morphological changes within the target organ except liver with cytoplasmatic hyperhydration and Kupffer cell activation.

Histological evaluation of potential target organs did not detect signs of inflammation or necrosis commonly associated with liver, spleen, central nervous system, or lung toxicity [20]. Increased activation of liver Kupfer cells witnessed transitory foreign body reactivity without any patho-histological detectable changes in other organs (Figure 10).

### 2.4. Magnetoemulsion Potential Therapeutic Effect Tested on ADSC

#### 2.4.1. In Vitro Wound Healing on ADSC Model

**Figure 11:**
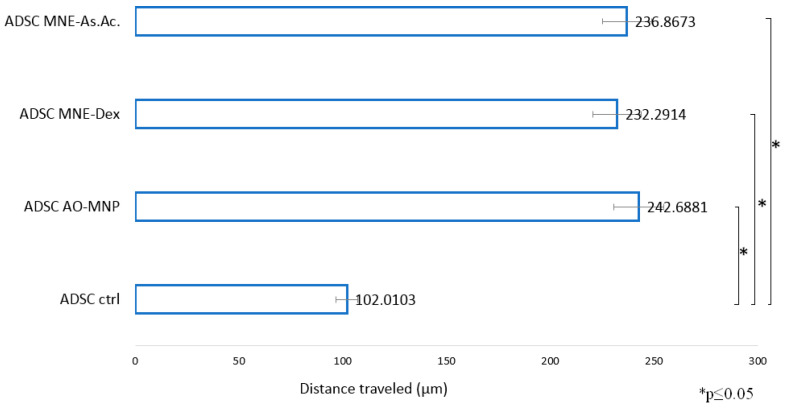
Cell mobility in an in vitro wound healing model. Time-lapse imaging over 24 h for each sample images taken at 10 min intervals. Images were processed using Digimizer 6 image analysis software. Ten cellular events were used for each speed calculation. * *p* ≤ 0.05.

Cell speed was calculated by tracking 10 cellular elements per sample by using Digimizer Version 6 image analysis software. In vitro motility was evaluated using a classical wound healing model ‘scratch test’ combined with time-lapse imaging over 24-h (one image every 10 min). A significant increased motility was observed for ADSCs treated with both types of MNEs compared to control (ADSCs cultivated without MNEs) (Figure 11).

#### 2.4.2. ADSC Chondrogenesis

**Figure 12:**
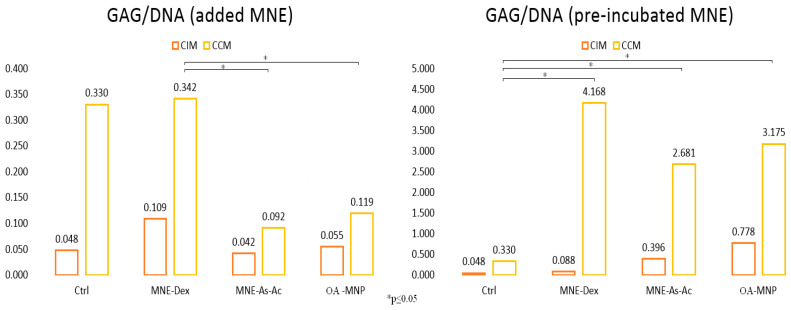
Quantitative evaluation of glycosaminoglycan (GAG) deposition/cell/pellet in ADSC demonstrating significant GAG deposition by ADSC pretreated with MNE before pelleting. * *p* ≤ 0.05.

Chondrogenic conversion of ADSC treated with an MNE suspension was assessed quantitatively by means of the amount of glycosaminoglycan (GAG) deposition by pelleted cells grown in complete (CCM) or incomplete (ICM) chondrogenic media (with or without the addition of TGF β3). The calculated amount of GAG/pellet obtained using DMMB colorimetric assay was normalized to DNA content/cell, considering that each cell has approximatively 6 pg DNA. MNEs for this test were delivered to ADSC, either after pelleting or 24–48 h before harvesting cells for the formation of a pellet. In the first case, MNE suspensions were added to the chondrogenic test tubes, while in the latter they were added to ADSC while still in 2D culture. The results indicated significantly increased GAG deposition by ADSC pretreated with MNEs before pelleting for both MNEs-Dex and MNEs-As.Ac. In the case of MNEs added after pelleting, GAG amount in non-treated ADSC and MNEs-Dex was found to be similar, while MNEs-As.Ac. derived significantly less GAG/pellet (Figure 12). Remarkably, while the amount of GAG/pellet for non-treated control ADSC remained very similar in both treatment protocols, GAG/DNA ratio was 13 times higher for MNEs-Dex in the pre-treatment group and 20 times higher for MNEs-As.Ac. compared to the situation when MNEs were added after pelleting.

## 3. Discussion

Here we report a relatively straightforward method of obtaining nanosized MNEs with long-term stability, magnetic properties, and high biocompatibility for carrying dexamethasone or ascorbic acid. A combination of low-energy (rapid injection) and high-energy (ultrasonication) methods was used to prepare MNEs (O/W) containing dexamethasone or ascorbic acid of nanometer sizes (below 200 nm). The method is highly reproducible and can be up-scaled and could be easily used for manufacturing large batches [24]. One of the most significant parameters of the nanoemulsions used in the biomedical field consists in stability. Nanoparticles that display a zeta potential of more than ±20 mV are generally considered stable [25,26]. Consequently, the zeta potential of the MNEs-Dex and MNEs-As.Ac. tested here reveals adequate stability, confirmed by the macroscopical appearance up to 5 months (Figure S1). Magnetic measurements demonstrated that the MNEs preserve superparamagnetic behaviors of the OA-MNPs, a fact that could be used further for guided delivery using magnetic fields and for their tracking using MRI.

Human primary ADSC exposed to increasing concentrations of MNE-Dex and MNE-As.Ac. (25, 50, 100, 125, 160 µg/mL) were found to preserve cell viability, even at high doses, as calculated relatively to MNPs content. We have previously demonstrated excellent viability of prepared, oleic, and palmitic acid coated proprietary MNPs [27]. Compared to other nanolipids (solid lipid nanoformulations (SLP) or nanostructured lipid carriers (NLP)), NEs with similar composition, size, and stability were shown to not induce in vitro or in vivo toxicity [28] and to remain stable when interacting with human blood plasma proteins [29]. The increased viability obtained in this study after long term exposure demonstrates, alongside excellent biocompatibility, the fact that ADSC are capable of proliferating in the presence of MNE with both cargo types. When delivered systemically (intraperitoneal route) in high doses to mice, MNE suspension with both cargo types did not induce inflammatory events as reflected in white blood cell count and CRP levels. No histological evidence of possible target organ (brain, cardiac muscle, spleen, kidney, cerebellum) impairment was found. The detected reactivity of liver Kupfer cells can be explained by the transitory and reversible changes encountered during foreign body reactions [30].

Several pilot experiments performed on fibroblast in vitro wound healing revealed an accelerated “gap closure” for fibroblasts treated with MNE (Figure S2, Supplementary Material). We therefore wanted to confirm if this feature persists on ADSC treated with MNEs-Dex and MNEs-As.Ac. Indeed, life imaging over 24 h confirmed that MNEs types significantly increase the distance traveled by distinct cellular elements in the culture dish compared to non-loaded controls. This indicates increased ADSC mobility and possible increased ability to migrate to sites of wound, inflammation, or regeneration. The established direct correlation between mesenchymal stem cells in vitro motility and regenerative potential supports this observation [31], which needs to be further confirmed in vivo.

The most remarkable finding was the fact that ADSCs not only preserve but significantly enhance chondrogenic conversion in the presence of MNEs. The results, however, depended on the timing and modality of exposure. When added after cell pelleting, MNEs-Dex significantly increased chondrogenic conversion compared to MNEs-As.Ac.; however, it remaining at similar levels with non-treated ADSCs. This can possibly be explained by the fact that a supplement of dexamethasone (a usual component of chondrogenic media) was released and acted in increasing GAG deposition. Dexamethasone was previously found to promote MSC chondrogenic differentiation [32], a process reportedly suppressed in dexamethasone-free cultures [33] acting by increasing proteoglycan and collagen type II content. Dexamethasone may act as a promoter of key masters of differentiation (such as Runx2 and Noggin) in a context-dependent manner [34]. In the case where ADSCs were preconditioned with MNEs, significantly higher chondrogenesis was detected for both MNEs cargo types compared to non-treated controls. In this case, the effect of engulfed MNPs probably influenced cell iron handling pathways and consequently increased chondrogenic conversion. Iron is reported as being essential for the cartilage formation required for successful ECM synthesis by chondrogenic cells. Reduced iron ions catalyze proline and lysine hydroxylation reactions, which are essential to the formation of mature collagen molecules [35]. Iron nanoparticles can provide a slow release of reduced iron ions, supporting chondrogenesis. During mesenchymal stem cell (MSC) chondrogenesis, transferrin was shown to be down regulated, while ferropontin increased compared to other differentiation pathways [36]. Moreover, combined ascorbic acid and iron supplementation (delivered as commercially available ferumoxytol) increased MSC chondrogenesis and improved the repair of Guinea minipig cartilage defects [37]. In this study, we found that magnetic nanoemulsions carrying dexamethasone and ascorbic acid consistently increase in vitro chondrogenic extracellular matrix deposition by ADSC compared to non-treated in situation cells pre-exposed to the respective formulation. This could be used as a modality of delivery of intra-articular dexamethasone that could exert chondroprotective effects in parallel with the anti-inflammatory ones. The addition of an MNE-As-Ac formulation could further improve chondroprotection. The good emulsion stability could allow for on-the-shelf formulations that could be customized upon patient needs in terms of the ratio between dexamethasone and ascorbic acid. Given the fact that iron oxide nanoparticles with different coatings [38], as well as dexamethasone and ascorbic acid, are already approved for clinical administration, the intra-articular delivery of nanoemulsions could be easily translatable to human and veterinary patients. The addition of potential remote controllable guiding and tracking using magnetic fields could be further developed.

## 4. Materials and Methods

All reagents and chemicals, if not otherwise mentioned, were purchased from Sigma-Aldrich (St. Louis, MO, USA) and Lonza (Basel, Switzerland).

### 4.1. Synthesis of MNEs

The proprietary MNPs were synthesized according to previously described methods [23]. To prepare MNEs, two types of lipids, oleic acid and tocopherol, using Tween 80 as a surfactant, were mixed in 1 mL ethanol (10:1:1 molar ratio) under ultrasonication for 5 min. To this solution, 5 mg of magnetic nanoparticles were added, before ultrasonication for 10 min. Further, 10 mg and 5 mg, respectively, of the biologically active compounds, dexamethasone/ascorbic acid, were dissolved in 1 mL of ethanol/deionized water, under ultrasound for 10 min. An amount of 1 mL of liposomes with embedded magnetic nanoparticles were added to this solution, before stirring for another 30 min at 70 °C. Finally, 100 µL of the resulting solution was quickly injected into 900 µL of deionized water, under ultrasound for 5 min, thus forming magneto-nanoemulsions with bioactive compounds: dexamethasone (MNEs-Dex) and ascorbic acid (MNEs-As.Ac.).

### 4.2. In Vitro Drug Release

The release assay of dexamethasone and ascorbic acid from magnetic nanoemulsions was carried out using a Lambda 35 UV/VIS Spectrometer (PerkinElmer, Waltham, MA, USA). For the release experiments, 4 mL of MNEs was introduced into the dialysis membranes (MWCO:14,000) and placed in a 50 mL tub containing 11 mL culture medium, under magnetic stirring at 37 °C. At predetermined time intervals, 0, 1, 3, 7, 16, 20, 24 h, for dexamethasone, and 0, 1, 2, 5, 16, 20, 22, 24 h, for ascorbic acid, 4 mL of the sample was withdrawn and placed in a quartz cuvette; the absorbance was measured at 242.7 nm for dexamethasone and at 264.6 nm for ascorbic acid. The cumulative drug release was calculated based on the standard curves for dexamethasone and ascorbic acid. The in vitro release experiments were performed in triplicate.

### 4.3. MNE Characterization

The hydrodynamic diameter and the surface charge of the MNEs were determined using a dynamic light scattering (DLS) instrument (Zatasizer Nano ZS, Malvern, UK). To evaluate MNE stability at room temperature over the long term, their size distribution at 2, 14, 90, and 150 days was measured using a dynamic light scattering method (DLS—Microtrac/Nanotrac 252, Montgomeryville, PA, USA). The magnetic measurements of OA-MNPs and MNEs were determined using a vibrating sample magnetometer (VSM) (LakeShore 7410, San Antonio, TX, USA) at room temperature (25 °C).

### 4.4. Cells—Human Primary ADSC

Cells were isolated from human adipose tissue samples obtained from the fresh lipoaspirate of donors undergoing elective cosmetic procedures, as previously described [17]. Samples were obtained after ethical board approval and patient informed consent. Briefly, the aspirate was washed three times with sterile phosphate buffered saline (PBS) with 2% antibiotic, and suspended in 0.1% collagenase (collagenase type I, Sigma-Aldrich). Digestion was carried out at 37 °C, 5% CO_2_ for 2–3 h; after incubation, the digest was centrifuged at 300× *g* for 5 min and filtered using 100 µm cell strainer. The pellets containing the SVF were resuspended in complete culture media CCM (DMEM with 10% FBS and 2% antibiotic) and plated in size-appropriate culture flasks. Cells in passage 3–5 were used for experiments.

### 4.5. In Vitro Cytotoxicity

In vitro cytotoxicity was performed after 24 h, 7 days, or 21 days cell-MNE (10% *v*/*v*) interaction using 5-dimethylthiazol-2-yl-2, 5-diphenyltetrazolium bromide (MTT) test (Vybrant MTT cell proliferation assay TermoFisher Scientific, Waltham, MA, USA) according to manufacturer’s instructions using dimethylsulphoxide (DMSO) as a dilution agent. We also investigated the cytotoxicity of MNEs containing MNPs at different concentrations (25, 50, 100, 125, 160 µg/mL) after 48 h of interaction with ADSCs. Absorbance was measured at 570 nm (Synergy HTX Multi-Mode Reader-Bioteck). Cell viability (CV) was calculated using the formula CV = 100 × (ODs − ODb)/(ODc − ODb). Ods = particle treated cell OD; ODb = blank (media only) OD; ODc = untreated cell optical density (OD).

### 4.6. Cellular Iron Uptake (Ferrozine Assay)

ADSC in passage 3 were plated in 24 well plates at 2 × 10^5^ cells/well. After 24 h, MNEs (10% *v*/*v*) were added to each well. After 2 days, cells were double washed with PBS to remove any extracellular iron content, and then the ferrozine assay was performed. Iron cellular content was calculated based on the spectrophotometric detection of iron in cell lysates normalized to the iron content of non-loaded counterparts, relative to cell number. A calibration curve was set up using FeCl_3_ standards (0–300 µM) in 10 mM HCl. The absorbance was measured at 550 nm on a microplate reader. The remaining wells were used for cell counting using a TC 20 Automated Cell Counter (BIO RAD). The intracellular iron concentration determined for the well was normalized against cell number/well.

### 4.7. Morphological Aspects of ADSC Loaded with MNE

Cell morphology and MNE upload was investigated under a fluorescent (optical) inverted microscope (EVOS FL Life Technologies, Carlsbad, CA, USA) after 48 h of ADSCs—MNEs interaction in complete media.

### 4.8. In Vitro Wound Healing Model

ADSCs were plated at 4 × 10^5^ cells in 36.5 mm diameter Petri dishes. For performing the in vitro wound healing assay (“the scratch test”), cells were removed along the Petri dish diameter with a 200-µL sterile pipette tip to form a cell-free area. The scratch-wounded cells were washed with PBS in order to remove any cell fragments before incubating in fresh complete media with 10% *v*/*v* MNEs for 24 h. Cells treated with complete medium were used as the control. Under a fluorescent inverted microscope, cells were observed using time lapse microscopy in a custom-made top-stage incubator. A software program (Digimizer 6) was used to process the images at intervals of 10 min.

### 4.9. Chondrogenesis

For chondrogenesis assays, 5 × 10^5^ ADSCs were pelleted in incomplete chondrogenic media (ICM) composed of DMEM (high glucose -HG), dexamethasone 1 mM, and ascorbic acid 2-P: 5 mg/mL L-Proline: 4 mg/mL ITS + supplement sodium pyruvate. After pelleting, ICM was changed with complete chondrogenic media (CCM = ICM plus TGF-β3 10 ng/mL). Chondrogenic pellets were kept in 15 mL polypropylene tubes at 37 °C, 5% CO_2_, and fed twice/week with CCM for 21 days.

### 4.10. Assessment of Chondrogenesis

After 21 days, pellets were digested using papain, and total amount of GAG glycosaminoglycans content was assessed using chondroitin-6-sulphate DMMB (1, 9 Dimethyl methylene blue) method. DMMB absorbance was read using a microplate reader. Total GAG amount per pellet was normalized to DNA content per pellet. The total DNA content was obtained using a DNA Quantitation Kit (Sigma Aldrich, Burlington, MA, USA), in which bisbenzimide, a fluorescent dye, binds with double stranded DNA so that when excited at 360 nm, the fluorescence emission at 460 nm increases proportionally to the amounts of DNA from the sample. The results are estimated using a standard curve.

### 4.11. In Vivo Biocompatibility

#### Animals

In our study, 24 mice of the BALB C strain purchased a month before the start of the experiment from the Cantacuzino Institute in Bucharest were used. The mice were 6–8-week-old nulliparous females, with a mean weight of 18.7 ± 1.2 g. Mice acclimation was performed under identical conditions of temperature (22 °C) and humidity (50%), with a light/dark cycle of 12 h. Each experimental group (*n* = 3) was housed in autoclavable polycarbonate cages of 1000 cm^2^. The animals had permanent access to water (ad libitum) (autoclavable bottles with drip system) and standardized food—Cantacuzino Institute—with the following composition: 23% protein, 10% fat, 50% carbohydrates, 8% crude fiber, and 9% vitamin-mineral premix, calcium carbonate, and phosphate, amino acids.

### 4.12. Experimental Design

Animal experiments were performed after institutional board approval. Mice were injected intraperitoneally with 300 µL of MNE dispersed in DMEM. At least three subjects per sample were used. Seven days after MNE injection, histological evaluation of potential target organs was performed. Blood was collected for routine hematological tests as well as for assessment of circulating inflammatory markers (see below).

The histology of target organs was performed as previously described. Briefly, after euthanasia, all specimens were fixed in 10% buffered formalin and embedded in paraffin with a tissue processor Leica TP1020 (Leica Microsystems GmbH, Wetzlar, Germany). Sections of 5 μm thickness were obtained with a Microtome SLEE CUT 6062 (SLEE Medical GmbH, Nieder-Olm, Germany), deparaffinized, and stained by the Masson trichrome techniques. Qualitative histology was performed from stained sections using a light microscope Leica DM 750 (Leica Microsystems GmbH, Germany) with an attached digital camera Leica ICC50 HD (Leica Microsystems GmbH, Germany), equipped with Leica Application Suit Software (LAS) version 4.2.

### 4.13. Hematology

Hematology was performed using a n Abaxis HM5 Vet Scan automated hematological analyzer, based on highly reproducible impedance technology. Blood samples were collected in vacutainers containing EDTA (directly from the cardiac cavity), an anticoagulant substance known to not interfere with hematological parameters. The samples were analyzed immediately after their collection. The following parameters were determined for each: white blood cells (WBC), lymphocytes (LYM), monocytes (MON), neutrophils (NEU), eosinophils (EOS), basophils (BAS), red blood cells (RBC), hemoglobin (HGB), hematocrit (HCT), mean corpuscular volume (MCV), mean corpuscular hemoglobin (MCH), mean corpuscular hemoglobin concentration (MCHC), red cell distribution width (RDWs), red cell distribution width % (RDWc), platelets (PLT), platelet (PCT), mean platelet volume (MPV), platelet distribution width (PDWs), and platelet distribution width % (PDWc).

### 4.14. Assessment of Circulating Inflammation Markers (C Reactive Protein-CRP)

Serum samples were used for the detection of circulating CRP using Mouse C Reactive Protein SimpleStep ELISA Kit (abcam ab222511, Cambridge, UK), as per the manufacturer instructions. Briefly, 50 µL of standards and samples are added to each well in triplicate, followed by the addition of 50 µL of the antibody mix. After 1 h of incubation at room temperature, the wells are washed multiple times to remove the unbound material. An amount of 100 µL of the TMB development solution is added to each well and during 20 min of incubation is catalyzed by HRP, generating blue coloration. This reaction is then stopped by the addition of 100 µL stop solution, completing any color change from blue to yellow. The signal is generated proportionally to the amount of bound analyte, and the intensity is measured at 450 nm. The serum samples used were diluted 1:400; results are estimated using the standard curve generated using the samples provided by the manufacturer.

### 4.15. Statistical Analysis

The differences were assessed with the unpaired t test, which was performed using GraphPad Prism version 9.2.0 for Windows, GraphPad Software, San Diego, CA, USA, www.graphpad.com (accessed on 21 April 2023). Two-tailed *p* values were calculated, and the differences were considered statistically significant when *p* < 0.05.

### 4.16. Ethical Implications

The study was conducted in accordance with the 2019 consolidated version of the Directive 2010/63/EU legal regulations on the protection of animals used for scientific purposes and with the approval of the Ethics Committee of Iasi University of Life Sciences (IULS) nr 799/29.05.2023.

## 5. Conclusions

We have described the synthesis of a potential on-the-shelf drug delivery platform based on magnetic nanoemulsions tested as a carrier for common anti-inflammatory drug dexamethasone and ascorbic acid. The relatively simple method of fabrication and the use of already approved drugs for clinical use could allow a rapid translation to a potential customized intra-articular therapy for the treatment of OA. Here we have demonstrated their excellent in vitro and in vivo biocompatibility and the in vitro potential for increasing ADSCs regenerative potential and chondrogenesis. Further in vivo studies will be needed to ascertain their ability in wound healing and OA models. The significant increase in ADSC migratory potential and chondrogenesis found in this study demonstrate that MNEs carrying dexamethasone could be used for their already approved role of mitigating OA symptoms while exerting a protective role on the cartilage layer. A slow release of ascorbic acid could further support cartilage regeneration.

## Figures and Tables

**Table 1:** Physical characteristics of magnetic nanoparticle coated with oleic acid and magnetic nanoemulsions carrying dexamethasone and ascorbic acid (size/diameter, polydispersity index, and zeta potential). NE = nanoemulsion; MNE = magnetic nanoemulsions; MNE-Dex = magnetic nanoemulsions with dexamethasone; MNEs-Ac.As = magnetic nanoemulsions with ascorbic acid.

Physical Characteristics	OA-MNPs	MNEs-Dex	MNEs-As.Ac.
Diameter (nm)	116.63	186.25	200
Polydispersity index	0.22	0.36	0.26
ζ-potential (mV)	−148.7	20.69	20.17

**Table 2:** In vivo biocompatibility test after intraperitoneal injection of MNE. RBC = red blood cells; WBC = white blood cells; HCT = hematocrit; HGB = hemoglobin; MCV = mean corpuscular volume; MCH = mean corpuscular hemoglobin; MCHC = mean concentration of hemoglobin; RDWc = red blood cell distribution width; PLT = platelet.

Sample	RBC (10^12^/L)	HGB (g/dL)	HCT (%)	MCV (fL)	MCH (pg)	MCHC (g/dL)	RDWc (%)	RDWs (%)	PLT (10^9^/L)
MNP OA 80 µg/mL	8.25 ± 0.55	13.55 ± 0.93	42.45 ± 3.65	51.50 ± 3.34	11.81 ± 0.81	25.84 ± 2.62	19.32 ± 1.54	38.3 ± 6.22	312.48 ± 18.41
MNP OA 160 µg/mL	8.60 ± 0.24	12.45 ± 0.53	41.91 ± 3.87	52.13 ± 1.81	12.14 ± 0.45	25.52 ± 1.52	19.26 ± 0.67	38.65 ± 5.12	321.42 ± 23.16
MNE-Dexa-80 µg/mL	8.46 ± 0.13	13.13 ± 0.67	42.13 ± 2.71	52 ± 2.72	12.45 ± 0.18	28.55 ± 2.34	19.22 ± 1.14	38.37 ± 6.38	341.23 ± 14.61
MNE-Dexa-160 µg/mL	9.46 ± 0.32	14.43 ± 0.88	43.45± 1.41	52 ± 1.89	12.15 ± 0.34	27.37 ± 2.87	17.91 ± 1.21	37.82 ± 5.69	323.26 ± 18.56
MNE-As.Ac 80 µg/mL	8.81 ± 0.42	14.51 ± 0.67	42.10 ± 3.23	50 ± 3.56	12.33 ± 0.42	28.83 ± 1.58	19.63 ± 0.28	38.34 ± 5.54	310.31 ± 34.87
MNE-As.Ac 160 µg/mL	8.70 ± 0.48	13.65 ± 0.14	43.06 ± 1.65	54 ± 3.11	12.11 ± 0.32	24.36 ± 2.83	18.15 ± 0.77	38.31 ± 6.43	296.42 ± 39.85
DMEM 300 µL	9.19 ± 0.18	13.80 ± 0.98	42.88± 2.84	53 ± 2.21	12.38 ± 0.41	28.18 ± 1.29	19.11 ± 0.47	38.33 ± 5.31	291.22 ± 28.48
Saline 300 µL	9.54 ± 0.36	14.35 ± 0.32	43.66± 2.14	54 ± 2.76	12.24 ± 0.45	28.44 ± 1.56	18.94 ± 1.87	39.12 ± 4.87	301 ± 26.26

## Data Availability

Data regarding in vivo and in vitro experiments are available from corresponding authors by reasonable request.

## Supplementary Materials

The supporting information can be downloaded at: https://www.mdpi.com/article/10.3390/ijms241511916/s1.
