# Supplementary material for: Magnetic Nanoemulsions for the Intra-Articular Delivery of Ascorbic Acid and Dexamethasone
Source: Int J Mol Sci. 2023 Jul 25;24(15):11916. doi: 10.3390/ijms241511916 (PMC10419142; doi:10.3390/ijms241511916)
Supplement: Supplementary file 1 [file ijms-24-11916-s001.zip › ijms-2521203-supplementary.pdf]

LOT	WBC (10^9/l)	NEU (10^9/l)	LIMF (10^9/l)	MONO (10^9/l)	EOS (10^9/l)	BASO (10^9/l)	NEU (%)	LIMF (%)	MONO (%)	EOS (%)	BASO (%)
**MNP OA 80 µg/ml**	6.3	0.92	5.32	0.06	0	0	14.6	84.44	0.95	0	0
**MNP OA 80 µg /ml**	6.515	1.03	5.405	0.08	0	0	15.76	83.015	1.215	0	0
**MNP OA 160 µg /ml**	6.49	1.34	5.04	0.1	0	0	20.76	77.63	1.6	0	0
**1Bs2**	6.46	0.95	5.4	0.1	0	0	14.75	83.64	1.61	0	0
**MNP OA 80 µg /ml**	6.95	1.31	5.42	0.2	0	0	18.94	78.05	3.01	0	0
**MNE-Dexa-80 µg /ml**	6.55	0.95	5.41	0.19	0	0	14.52	82.59	2.9	0	0
**4Bs1**	6.28	1.28	4.92	0.07	0	0	20.5	78.39	1.11	0	0
**MNE-Dexa-160 µg /ml**	6.04	0.95	4.98	0.1	0	0	15.83	82.52	1.65	0	0

Table S1 White blood cell profile in mice serum sample after intraperitoneal injection of MNE. WBC=white blood cells; NEU=neutrophiles, LIMF- lymphocytes, EOS- eosinophile, BASO= basophile MONO= monocytes

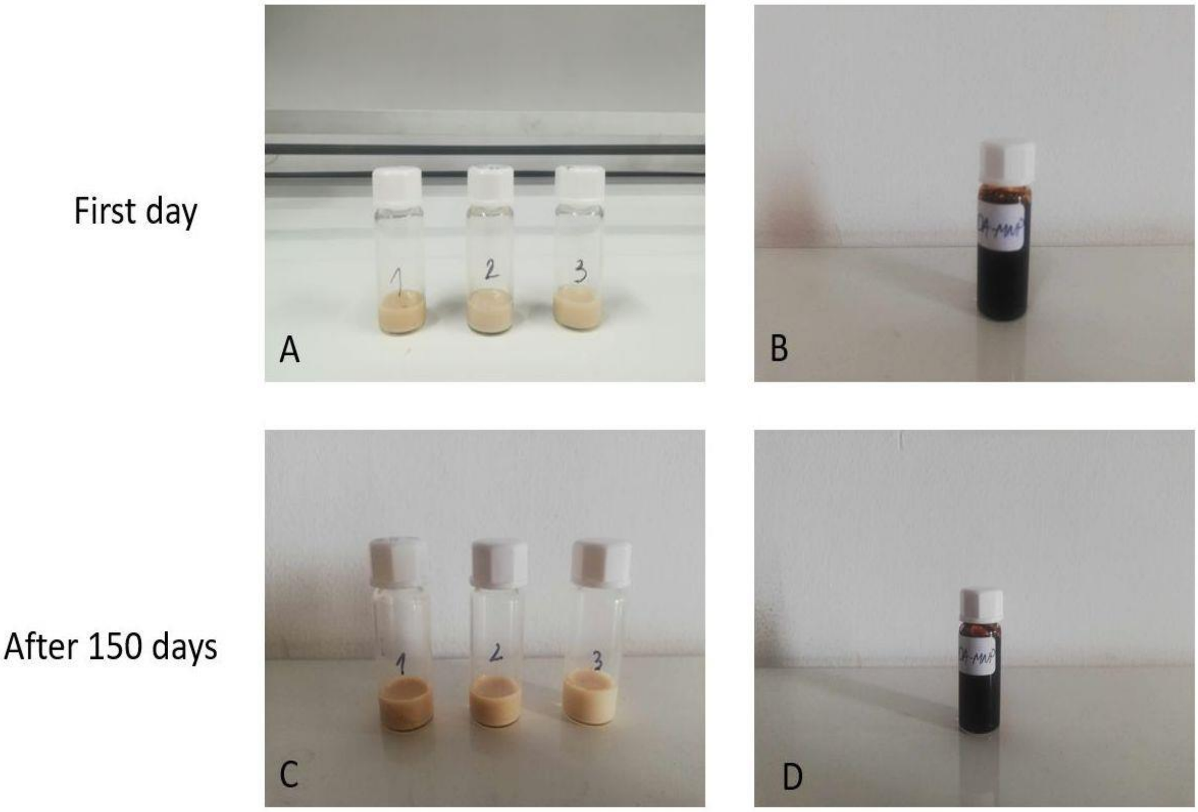

Figure S1. Macroscopical aspect of MNE demonstrating long term on the shelf stability in emulsion first day after preparation: A=MNE, MNE−Dex, MNE−As.Ac B=MNP-OA; 150 days after preparation: C= MNE , MNE−Dex, MNE−As.Ac; D=MNP-OA

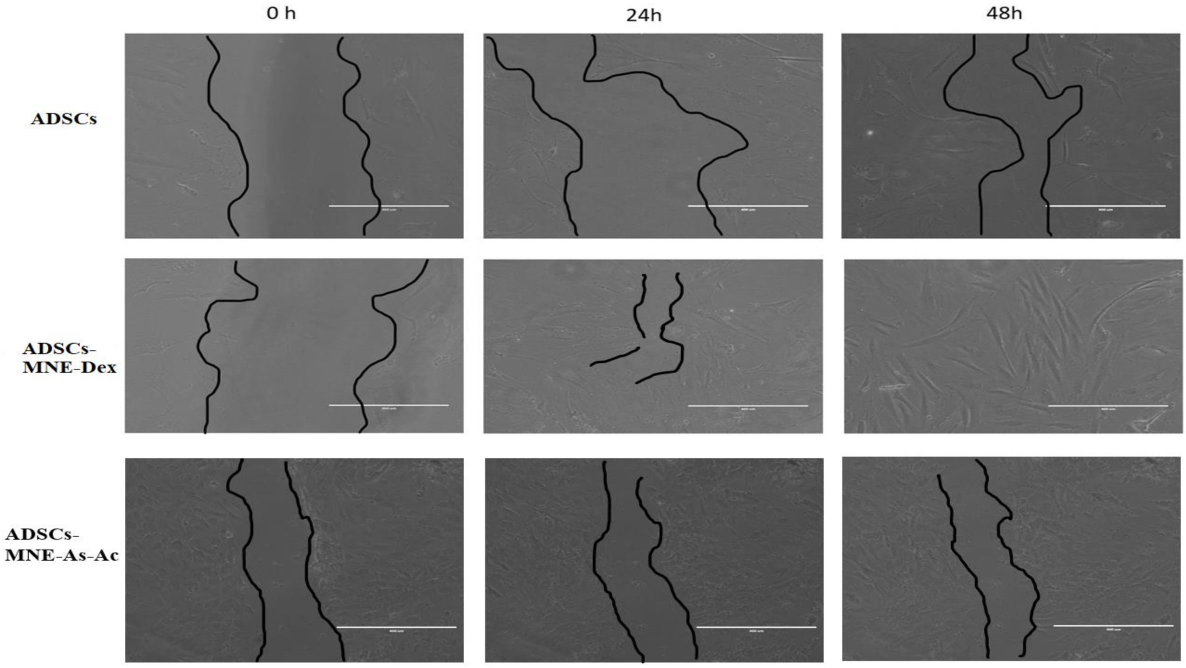

Figure S2: Wound healing model “scratch test” gap closure at 48 hours ADSC; ADSC with MNEs−Dex; ADSC with MNEs−As.Ac.
